# The quality of life of men experiencing infertility: a systematic review

**DOI:** 10.1186/s12889-024-18758-6

**Published:** 2024-05-05

**Authors:** Zahra Kiani, Masoumeh Simbar, Farzaneh Rashidi, Farid Zayeri, Homayoon Banaderakhsh

**Affiliations:** 1 Midwifery and Reproductive Health Research Center, Shahid Beheshti University of Medical Sciences, Tehran, Iran; 2 Midwifery and Reproductive Health Research Center, Department of Midwifery, School of Nursing and Midwifery, Shahid Beheshti University of Medical Sciences, Tehran, Iran; 3 Department of Midwifery, School of Medicine, North Khorasan University of Medical Sciences, Bojnurd, Iran; 4 Proteomics Research Center, Department of Biostatistics, Faculty of Allied Medical Sciences, Shahid Beheshti University of Medical Sciences, Tehran, Iran; 5 Department of Anesthesia and Operating Room, School of Nursing and Midwifery, Shahid Beheshti University of Medical Sciences, Tehran, Iran

**Keywords:** Quality of life, Men, Infertility, Systematic review

## Abstract

**Background:**

Men experiencing infertility encounter numerous problems at the individual, family, and social levels as well as quality of life (QOL). This study was designed to investigate the QOL of men experiencing infertility through a systematic review.

**Materials and methods:**

This systematic review was conducted without any time limitation (Retrieval date: July 1, 2023) in international databases such as Scopus, Web of Science, PubMed, and Google Scholar. The search was performed by two reviewers separately using keywords such as QOL, infertility, and men. Studies were selected based on inclusion and exclusion criteria. The quality of the articles were evaluated based on the Newcastle-Ottawa Scale. In the initial search, 308 studies were reviewed, and after removing duplicates and checking the title and abstract, the full text of 87 studies were evaluated.

**Results:**

Finally, 24 studies were included in the final review based on the research objectives. Based on the results, men’s QOL scores in different studies varied from 55.15 ± 13.52 to 91.45 ± 13.66%. Of the total reviewed articles, the lowest and highest scores were related to mental health problems and physical dimensions, respectively.

**Conclusion:**

The reported findings vary across various studies conducted in different countries. Analysis of the factors affecting these differences is necessary, and it is recommended to design a standard tool for assessing the quality of life of infertile men. Given the importance of the QOL in men experiencing infertility, it is crucial to consider it in the health system. Moreover, a plan should be designed, implemented and evaluated according to each country’s contex to improve the quality of life of infertile men.

## Introduction

Defined as the absence of pregnancy after one or two years of unprotected sexual intercourse (without the use of contraceptive methods) [1], infertility is recognized as both a medical and social issue [2]. Based on the latest Word Health Organization (WHO) report in 2023, the pooled lifetime and period prevalence of infrtility are reported as 17.5% and 12.6%, respectively [3]. In this regard, male factors play a role in 50% of infertilities [4].

Complicated treatment protocol, difficult treatment process, semen analysis, multiple ultrasounds, invasive treatments, long waiting lists, and high financial costs for the clients who seek assisted reproductive techniques have been described as psychological stresses for infertile couples [5, 6]. Moreover, the diagnosis and treatment of infertility can have negative impact on the frequency of sexual intercourse, self-esteem, and body image [5]. However, these men usually tend to suppress or deny their problems which may diminish their quality of life (QOL) over time [7]. This decreased QOL, in turn, can have a detrimental effect on their response to treatment [8].

The function of infertile people is under the influence of society, family, and the society culture. In many societies, infertility is primarily viewed as a medical problem, often neglecting its individual and social dimensions [9]. In other words, despite having the right attitude toward infertility, infertile people sometimes cannot adapt to the problem. Thus, non-compliance during the behavioral process may lead to additional problems and impair one’s QOL [10].

The WHO describes the QOL as people’s perspective of their life circumstances in terms of the cultural systems and standards of their environment, and how these perspectives are associated with their objectives, prospects, ideals, and apprehensions [11]. Recently, the QOL of men experiencing infertility as a main subject has been carefully considered by health investigators. Furthermore, because of men’s essential role in future phases of life, their QOL can significantly affect their health at both individual and societal levels [12].

Given the significance of QOL, its precise measurement is substantially important. In this regard, various tools have been designed and used in studies to examine this concept. A systematic study used the World Health Organization Quality Of Life )WHOQOL), 36-Item Short Form Survey (SF-36 ), and general QOL questionnaires. Based on the results, the QOL of men experiencing infertility was reported to be low in two studies that had used the SF-36 questionnaire. By contrast, the QOL of these men was high in a study that used the WHOQOL questionnaire. It was noted in this systematic review that although infertility has a negative effect on the mental health and sexual relationships of couples, there is no consensus regarding its effect on the QOL of infertile couples [13].

In Almutawa et al.‘s systematic review and meta-analysis 2023, it has been shown that the psychological disturbances in infertile women are higher than in men, and this difference in couples needs further investigation [14]. Chachavomich et al. 2010 showed that women’s quality of life is more affected by infertility than men study, which was a systematic review [12], . This study was conducted 14 years ago and due to the increase in the number of articles in this field, it needs to be re-examined.Given that no systematic review had been conducted to address the QOL of men experiencing infertility and considering the significance of this issue in therapeutic responses, this study examined the QOL of men experiencing infertility in the form of a systematic review.

## Methods

### Search strategy

To search and review the studies, reputable international databases and sites such as Scopus, Web of Science, PubMed, and Google Scholar were used. The search was performed using keywords such as QOL, infertility, and men (Table 1), without time limitation (Retrieval date: July 1, 2023), and using AND and OR operators, and specific search strategies were used for each database.

**Table 1:** The search strategy keywords

Category 1	Category 2	Category3
[‘Quality of Life’, ‘Health-Related Quality of Life’]	[‘Infertility’, ‘Sterility, Reproductive’, ‘Sterility’, ‘Reproductive Sterility’, ‘Subfertility’, ‘Sub-Fertility’]	[‘Male’, ‘Man’, ‘Boy’]

The search strategy of PubMed, Web of Science, and Scopus databases is as follows:

### Pubmed (retrieval date: July 1, 2023)

Male [tiab] OR Males [tiab] OR Men [tiab] OR Man [tiab] OR Boy [tiab] OR Boys [tiab] AND Quality of Life [tiab] OR Health-Related Quality of Life [tiab] AND Infertility [tiab] OR Sterility OR Reproductive [tiab] OR Reproductive Sterility [tiab] OR Subfertility [tiab] Sub-Fertility [tiab].

### Web of science (retrieval date: July 1, 2023)

((TI=(male OR males OR man OR men OR boy OR boys)) AND TI=(Quality of Life OR Health-Related Quality of Life OR Health-Related Quality of Life)) AND TI=(Infertility OR Sterility OR Reproductive OR Reproductive Sterility).

### Scopus (retrieval date: July 1, 2023)

TITLE ( male OR males OR men OR man OR boy OR boys ) AND TITLE (quality AND of AND life OR health-related AND quality AND of AND life ) AND TITLE ( infertility OR sterility OR reproductive).

The method of presenting the article, describing the problem, data collection, data analysis, discussion, and conclusion of the findings were based on preferred reporting items for systematic reviews and meta-analyses (PRISMA) 2020 [15]. The reviews were conducted separately by two reviewers, and the third reviewer was also used in case of disagreement between them.

## Inclusion and exclusion criteria

Those studies with the following criteria were included in the review: (1) Observational studies; (2) Cross-sectional data from longitudinal studies; (3) Using valid tools for measuring the QOL; (4) Studies conducted on men of infertile couples (by men experiencing infertility we mean those men whose unprotected sexual intercourse during the past year did not lead to any pregnancy); (5) Minimum sample size of 30 subjects; (6) Subjects with no chronic disease, and (7) those men of infertile couples who were within the diagnostic process for infertility and before starting infertility treatment. The search and review process for this study were conducted in English, and there were no restrictions imposed on the inclusion of open-access studies.

Exclusion criteria included: (1) Case report studies; (2) Review studies; (3) Animal studies; (4) Studies on mental syndromes; (5) Studies not written in English; (6) Lack of access to the full text of the article, and (7) Unrelated reports.

### The patient, intervention, comparison, outcome, and study design (PICOS)

PICOS model was used to help break down the searchable elements of the research question into (P) participants: men experiencing infertility (primary or secondary infertility) (I) intervention/exposure: not applicable; (C) control group: not applicable; (O) outcomes: evaluate infertile men’s QOL, which was measured using standard tools such as general or specific QOL questionnaire and (S) study type: Observational studies and Cross-sectional data from longitudinal studies.

## Data extraction

The two reviewers independently reviewed the titles and abstracts of the articles following the inclusion criteria, and the studies which did not have the required criteria were excluded. Then, the full text of the articles with inclusion criteria was reviewed and if appropriate, they were included in the study.

Required information, including authors’ names, year of publication, research location, sample size, QOL score, type of tool, type of infertility, mean age of men, and duration of infertility, were extracted from the studies.

### Outcome measurement

The main outcome of this study was to evaluate QOL of men experiencing infertility, which was measured using standard tools such as a general or specific QOL questionnaire.

## Quality evaluation

The Newcastle-Ottawa Scale checklist was used to assess the quality of nonrandomized studies in meta-analyses [16]. This checklist consists of 5 parts that are representativeness of the sample, sample size, non-respondents, ascertainment of anxiety, and quality of descriptive statistics reporting. Each part gets a score of zero and one. Given the fact that the checklist has 5 items, the minimum, and maximum scores are 0 and 5, respectively. Then, studies were divided into high- and low-risk groups if their scores were ≤ 3 and more than 3 [16]. The quality assessment in this study was performed by two reviewers independently, and in case of disagreement between them, the third reviewer was asked to help. The coefficient of agreement of 0.7 and more among the reviewers was acceptable.

## Ethical consideration

Ethics approval was obtained from the Ethics Committee, Faculty of Pharmacy and Nursing.

Midwifery, Shahid Beheshti University (Ethical code: IR.SBMU.PHARMACY.REC.1400.214). All methods were carried out in accordance with relevant guidelines and regulations.

## Results

**Fig. 1:**
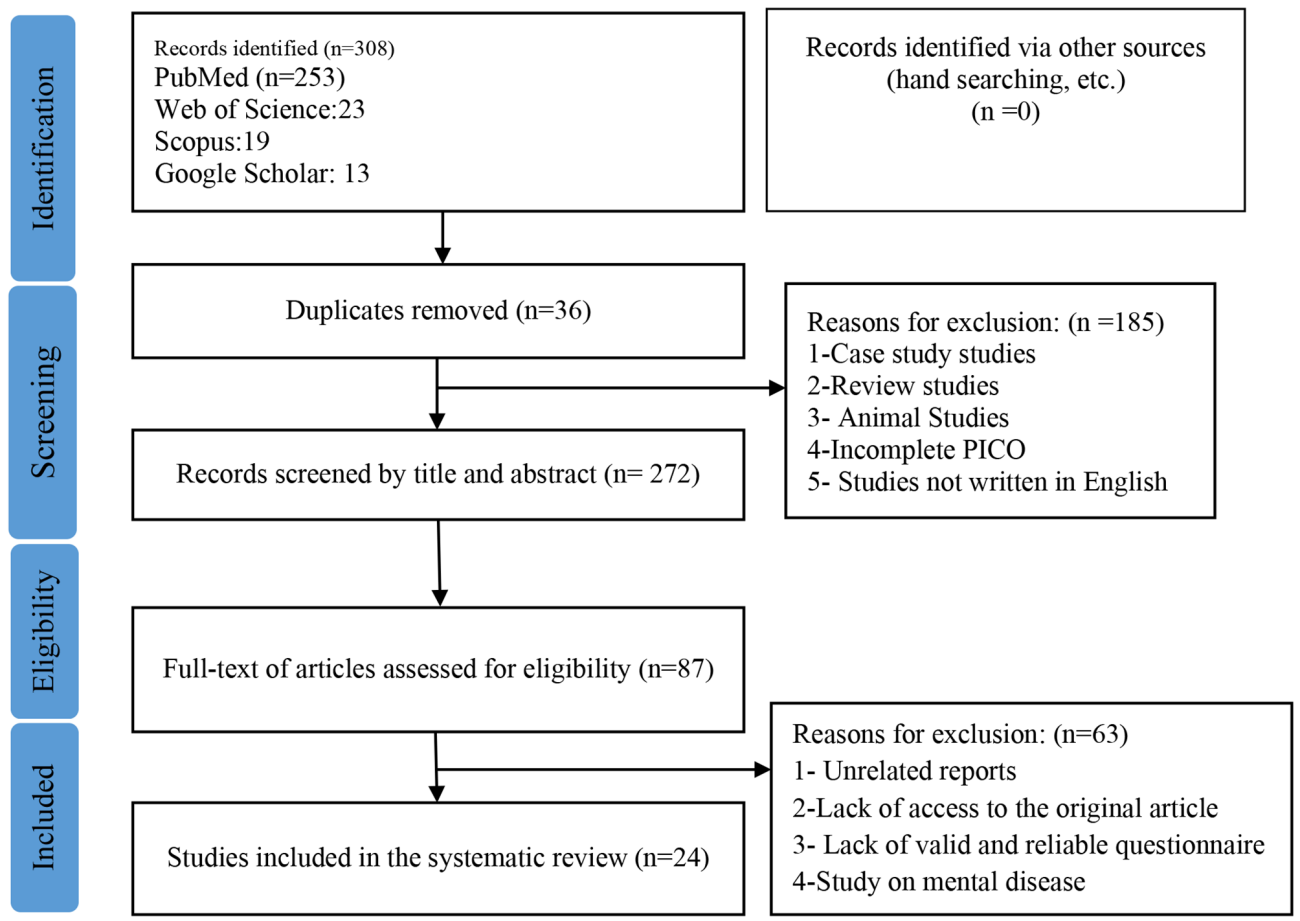
Flowchart for selection of studies

After reviewing the title, abstract, and text of the articles in different stages (Fig. 1), finally, 24 articles were reviewed based on the inclusion criteria and research objectives and the coefficient of agreement among the reviewers was K = 0.81 (Table 2).

**Table 2:** Characteristics of the studies selected

ID	Authors, year (ref)	countries	Sample size	Age (Y) (mean ± SD)	Mean years of infertility (mean ± SD)	Total Scores%	Low domain	High domain	Education level	Marital relationship	Family income	Income level contery based on world bank report	Type of Tools	OTTAWA score ˃ 3 low risks and ≤ 3 high risk
1	Andrei et al., 2021 [17]	Italy	38	38.2 ± 4.5	NA	78.6 ± 12.1	Relational	Mind-body	Positive and significant relationship	NA	Positive and significant relationship	High	FertiQoL	3.5
2	Warchol-Biedermann [18]	Poland	225	30.24 ± 4.29	NA	81.9 ± 7.5	Emotional	Environment	Positive and significant relationship	NA	Positive and significant relationship	High	FertiQoL	4.5
3	Asazawa et al. [19]	Japan	31	36.8 ± 7.0	2.9 ± 1.9	64.3 ± 9.5	Social	Mind-body	NA	NA	NA	Low and middle	FertiQoL	3.5
4	Cusatis et al. [20]	USA	67	NA	NA	79.7 ± 14.7	Relational	Mind-body	Positive and significant relationship	NA	no significant relationship	High	FertiQoL	3.5
5	Asazawa et al. [21]	Japan	321	37.9 ± 5.2	3.1 ± 3	69.9 ± 10.8	NA	NA	NA	NA	NA	Low and middle	FertiQo^1^	4
6	Shahraki et al. [22]	Iran	87	34.4 ± 8.2	6.8 ± 5.5	89.2 ± 12.9	limitations in social activities because of physical or emotional problems	limitations in physical activities because of health problems	Positive and significant relationship	NA	Positive and significant relationship	Low and middle	SF-36	3.5
7	Jahromi et al. [23]	Iran	501	32.66 ± 5.65	7.2 ± 4.1	62.1 ± 9.8	Social	Mind-body	Positive and significant relationship	NA	Positive and significant relationship	Low and middle	FertiQoL	4.5
8	Goker et al. [24]	Turkey	150	34.4 ± 5.9	3.8 ± 3.3	74.0 ± 13.6	Social	Mind-body	Positive and significant relationship	NA	Positive and significant relationship	High	FertiQoL	4.5
9	Kim et al. [25]	South Korea	121	36.7 ± 5.8	8.3 ± 3.6	91.45 ± 13.66	Social	Mind-body	Positive and significant relationship	Positive and significant relationship	Positive and significant relationship	High	FertiQoL	3.5
10	Casu et al. [26]	Brazil	152	38.21 ± 6.2	NA	NA	psychological health	environmental health	NA	NA	NA	High	WHOQOL-BREF	3.5
11	Maroufizadeh et al. [27]	Iran	180	30.54 ± 5.39	4.83 ± 3.61	72.89 ± 15.94	Social	Mind-body	Positive and significant relationship	NA	NA	Low and middle	FertiQoL	3.5
12	Zurlo et al. [28]	Italy	250	34.0 ± 3.85	3.0 ± 2.4	55.15 ± 13.52	NA	NA	Positive and significant relationship	NA	NA	High	FertiQoL	4.5
13	Madero et al. [29]	Spain	201	41.6 ± 5.6	NA	78.7 ± 13.2	Emotional	Environment	NA	NA	NA	High	FertiQoL	3.5
14	Agostini et al. [30]	Italy	85	39.8 ± 4.99	NA	81.48 ± 19.95	limitations in usual role activities because of emotional problems	vitality (energy and fatigue)	NA	NA	NA	High	SF-36	3.5
15	Kissi et al. [31]	Tunisia	100	38.74 ± 4.6	5.19 ± 4.62	81.56 ± 12.06	NA	NA	No significant	NA	No significant	Low and middle	SF-36	3.5
16	Huppelschoten1 [32]	Netherlands	670	35.0 ± 5.6	NA	Median:80.2 (35.4–100/0)	Relational	Mind-body	Positive and significant relationship	NA	NA	High	FertiQoL	4.5
17	Onat and Beji [33]	Turkey	58	36.2 ± 8.6	4.8 ± 2.1	71.96 ± 15.11	social relationships	Physical health	Positive and significant relationship	Positive and significant relationship	NA	upper-middle-	WHOQOL-BREF	3.5
18	Herrmann et al. [34]	Germany	199	35.6 ± 3.7	4.0 ± 5.1	70.41 ± 1.90	psychological health	Physical health	NA	Positive and significant relationship	NA	High	WHOQOL-BREF	3.5
19	Bolosy et al. [35]	Turkey	107	NA	NA	78.37 ± 2.14	social relationships	Physical health	No significant	NA	No significant	upper-middle	WHOQOL-BREF	3.5
20	Chachamovich et al. [36]	Canadá	162	36.1 ± 7.9	NA	NA	limitations in usual role activities because of emotional problems, social relationships	limitations in physical activities because of health problems, physical health	Positive and significant relationship	Positive and significant relationship	NA	High	SF-36 WHOQOL-BREF	3.5
21	Chachamovich et al. [37]	Brazil	150	36.1 ± 7.9	5.7 ± 3.6	73.9 ± 14.3	NA	NA	NA	NA	NA	High	WHOQOL-BREF	4.5
22	Drosdzol and Skrzypulec [38]	Poland	260	31.4 ± 4.7	3.2 ± 4.1	66.98 ± 5.6	limitations in usual role activities because of emotional problems	environmental health	Positive and significant relationship	Positive and significant relationship	Positive and significant relationship	High	SF-36	4.5
23	Rashidi et al. [39]	Iran	514	35.9 ± 0.6	7.5 ± 3.2	68.32 ± 17.7	general mental health (psychological distress and well-being	limitations in physical activities because of health problems	Positive and significant relationship	NA	NA	Low and middle	SF-36	4.5
24	Ragni et al. [40]	Italy	1000	39.02 ± 4.6	5.6 ± 2.7	83.55 ± 17.18	limitations in usual role activities because of emotional problems	limitations in physical activities because of health problems	NA	NA	NA	High	SF-36	4.5

FertiQoL: The Fertility Quality of Life tool, SF-36: The Health Survey Short Form, WHOQOL-BREF: World Health Organization Quality of Life Instruments, NA: Not Report

The smallest and largest sample size were 30 [19] and 1,000 [40], respectively. Seven studies were conducted in low- and middle-income countries, two studies in upper-middle-income, and 15 studies in high-income countries. High-income countries had a higher quality of life score compared to low- and middle-income countries countries. In all studies, QOL scores were calculated based on 100, and the highest score (91.45 ± 13.66%) obtained from the Fertility quality of life (FertiQoL) questionnaire in South Korea as a high-income country [25]. Most of the studies showed that education, family income and proper marital relations improved the quality of life of infertile men. Out of 24 reviewed articles, 12 articles used the FertiQoL questionnaire, 7 articles SF-36, and 6 articles WHOQOL-BREF. One study [36] used SF-36 and WHOQOL-BREF questionnaires simultaneously.

Out of the total articles reviewed, the lowest scores were attributed to different domains. Accordingly, the lowest score in 11 articles was related to mental health problems, in 8 articles it was related to social problems, and 3 articles to communication problems.Some articles did not report the scores based on the dimensions. Based on the results, men’s QOL scores in different studies varied from 55.15 ± 13.52 to 91.45 ± 13.66%. In the total reviewed articles, the lowest and highest scores were related to mental health problems and physical dimensions, respectively.

In most of the studies using the FertiQoL questionnaire, it was observed that the lowest scores belonged to the social and communication dimensions. The FertiQoL questionnaire was developed and psychometrically evaluated in a survey study conducted in the United States. FertiQoL is a 36-item scale with Six dimension: (1) Emotional; (2) Mind-body; (3) Relational; (4) Social; (5) Environment; and (6) Treatment tolerability. A 5-point Likert scale (0–4) was used in the questionnaire, and the total score was between 0 and 100, where the higher the score, the better was the QOL [41]. This questionnaire has been translated into different languages in the world and has obtained the required validity (content, face, and construct) and reliability (with Cronbach’s alpha of 0.7–0.9) in different populations [42–45].

In the studies where the SF-36 and WHOQOL-BREF questionnaires had been used, the lowest scores belonged to the dimensions of limitations in usual role activities because of emotional problems and social relationships. On the other hand, the highest scores in the questionnaires were related to physical dimensions. The SF-36 questionnaire has been considered for clinical investigation, health policy assessments, and surveys. The 8 dimensions of this questionnaire are as follows: Restrictions in physical activities; Restrictions in social activities; Restrictions in standard role activities; Physical pain; General mental health; Restrictions in standard role activities; Vitality; and Common health perceptions. The final scores of the questionnaire are standardized based on 100 [46]. This questionnaire has been translated into different languages in the world and has obtained validity (content and face) and reliability (Cronbach’s alpha of 0.8–0.95) in different populations [47–52]. The 26-item version of WHOQOL-BREF was developed in the following four dimensions: physical health, mental health, social connections, and environmental health, and two items associated with common QOL and general health [53]. The questionnaire has been translated into different languages of the world and has obtained validity (content and face) and reliability (Cronbach’s alpha of 0.74–0.88) in different populations [54–57].

## Discussion

This systematic review study investigated the quality of life of infertile men. Based on the results, men’s quality of life scores in different studies varied from 55.15 ± 13.52 to 91.45 ± 13.66%. However, men’s quality of life scores was reported to be between 70 and 80% in the majority of the studies. As one of the health indicators with a combination of each person’s knowledge in different aspects of life and performance in human, work and social relations, quality of life is essentially important for the continuation of an optimal life and well-being of the individuals. Moreover, quality of life is strongly influenced by demographic, social, economic, and cultural variables, as well as the variables related to health and disease, and its measurement is, thus, substantially important [58]. Quality of life is a reflection of the desires, hopes, and expectations of individuals regarding their current and future life situation, and is influenced by factors such as age, personal and family characteristics, socio-economic status, and time [59].

In this systematic review, the lowest scores of men’s quality of life belonged to the psychological and emotional dimensions and then to the social and communication dimensions. Although the reviewed studies had used different tools, these tools were essentially similar in these dimensions, indicating the problems of men in these areas. Fertility is highly valued in most cultures and the desire for having a child is one of the human stimuli in the continuation of life. If efforts for fertility do not lead to success, they can have adverse effects on mental health as well as family and social relationships [60].

The reviewed studies indicated that education has a significantly positive effect on the quality of life of infertile men. Higher levels of education are associated with increased awareness and better decision-making abilities [25], and improved coping strategies for dealing with infertility-related challenges [38]. Infertile men with higher education are also more likely to seek treatment, and remain hopeful that treatment will improve their quality of life [28].

The results of most studies showed the positive and significant relationship between family income and quality of life.The costs of infertility treatment and the potential need for repeated treatment can lead to concerns and anxieties among men and reduce their quality of life [61]. If men have fewer concerns about the cost of treatment, they are more inclined to pursue infertility treatment. In the International Conference on Population and Development held in Cairo in 1994, addressing the issue of infertility was emphasized as an important health priority. However, it is unfortunate that infertility problems have been overlooked not only in developing countries but also at various levels of international health management [62].

The results of the study regarding the countries’ income showed that the quality of life score of men in infertile couples residing in low-income countries was lower compared to those in high-income countries. Current infertility policies in the treatment and distribution sector are uncoordinated, which has led to improper distribution of public and private centers in low- and middle-income countery [63]. This point of view is a kind of simplistic calculation of the problem of infertility that justifies the lack of public centers, inadwquate finantial sources, specialists and affordable treatment options [64], requireing serious attention and careful planning, especially in low- and middle-income countries.

The results of the studies showed that marital relationships have a positive and significant impact on the quality of life of infertile men. Sometimes, infertile men may experience a lack of sexual attraction, and due to irrational thoughts, they might abstain from having sexual relations with their partners or try to suppress their sexual desires. Sexual desire is a significant aspect of life that can affect the quality of life [65]. Some studies have indicated that the quality of marital relations is higher among infertile couples than the fertile ones, and infertility can bring couples closer together and encourage more open communication about their concerns and plans for the future [33, 66]. Further research is recommended to gain a deeper understanding in this area.

Infertility presents people with a new and challenging world [28]. In this regard, infertility is characterized as a long-term process that involves time-consuming treatments, fluctuations between hope and disappointment, loss of control over reproductive outcomes, inability to plan for future, and significant shifts in personal identity and worldview [28, 32, 63]. Long working hours, work-caused exhaustion, along with infertility, can exacerbate men’s problems. These problems affect their quality of life, though they may deny the problems [67].

Given the significance of quality of life, its accurate measurement is essentially important. In this regard, various tools have been designed to investigate this concept and have been used in several studies. The noteworthy point in this systematic review was the use of different measurement tools in various studies. In the majority of the studies, Boivin’s FertiQoL [41] was used as a specific tool for measuring the quality of life of infertile couples. Covering emotional, physical, communicational, social, environmental, and acceptability dimensions, this questionnaire has been designed for infertile couples and does not specifically assess the quality of life of infertile men. Other studies have used a general quality-of-life questionnaire (SF-36 and WHOQOL-BREF). WHOQOL questionnaire has been designed in 4 dimensions of physical health, psychological health, social relationships, and environmental health [53]. SF-36 questionnaire also has 8 dimensions of Limitations in physical activities because of health problems; 2) Limitations in social activities because of physical or emotional problems; 3) Limitations in usual role activities because of physical health problems; 4) Bodily pain; 5) General mental health (psychological distress and well-being); 6) Limitations in usual role activities because of emotional problems; 7) Vitality (energy and fatigue); and 8) General health perceptions [46]. The main drawback of these tools is that they ignore significant dimensions such as sexual and socio-economic dimensions which are important for certain groups including infertile men. Additionally, the other dimensions of the questionnaire are not sensitive enough to measure changes in the quality of life of people with various diseases [68].

Health researchers have recently paid much attention to the examination of the quality of life and the design of a questionnaire to measure this concept. This measurement can improve clinical decision-making, estimate healthcare in a particular population, perceive different health causes and consequences, and, finally, promote health policy. All of these objectives will be achieved in light of a specific tool in this regard. However, according to the review, no questionnaire has hitherto been designed to measure the quality of life in infertile men. Specific questionnaires for infertile couples or general quality of life questionnaires have been used in different studies. Given the concept of quality of life and its changes over time as well as the expansion of tool-making knowledge, there is a need to design specific tools to measure the quality of life of infertile men by using mixed methods. We hope that more attention will be given to this significant issue in future. Polit and Beck argue that one of the main applications of exploratory mixed methods is in instrument making. They maintain that when a new tool is developed to explain a health-related concept, the complexity of this concept must be carefully explained [69].

Furthermore, it seems that the concept of men’s quality of life needs more investigation and also this concept may change over time and impact on their life. Besides, the studies demonstrated specific concerns among infertile men such as decreased self-esteem, Fertility- related stress, masculinity identity, hiding the infertility problem, resistance to the treatment, and cost of treatment [70, 71]. These concerns could be the specific items for the infertile men-related quality of life questionnaire.

### Research limitations

The impossibility of meta-analysis was because of several limitations in the study: (1) Variety of tools and small sample size in each subgroup; (2) Inaccurate report of information; and (3) -heterogeneity of the studies. Other limitation in this systematic review was that the reviewed papers were confined to English literature; thus, it is possible that some relevant non-English language studies were missed.

### The systematic review strategies and solutions

The quality of life of men is one of the basic issues in their life. Assessing the quality of life of men should be done during the initial evaluation of infertility, and if necessary, interventions should be made to improve their quality of life. It is recommended that researchers, using qualitative-quantitative methods, first explain the concept of the QOL of men with infertility and then design and psychometrically evaluate the QOL tool for men experiencing infertility. Based on its context, each country should design a suitable program to improve the quality of life of men.

## Conclusion

The reported findings vary across various studies conducted in different countries. Analysis of the factors affecting these differences is necessary, and it is recommended to design a standard tool for assessing the quality of life of infertile men. Given the importance of the QOL in men experiencing infertility, it is crucial to consider it in the health system. Moreover, a plan should be designed, implemented and evaluated according to each country’s contex to improve the quality of life of infertile men.

## Data Availability

All data related to this review is included in the result section of the manuscript. If any further data is needed it can be accessible via the corresponding author on request.

## Abbreviations

WHO: World Health Organization
NOS: The Newcastle-Ottawa Scale
PRISMA: Preferred reporting items for systematic reviews and meta-analyses
NA: Not reported
SF-36: The Health Survey Short Form
WHOQOL-BREF: World Health Organization Quality of Life Instruments
FertiQoL: The Fertility Quality of Life tool
